# Mother and Child

**Published:** 1902-10

**Authors:** 


MOTHER AND CHILD. By EDWARD F. DAVIS, A. M., M. D., Professor of Obstetrics in the Jefferson Medical College; Professor of Obstetrics and Diseases of Infancy in the Philadelphia Polyclinic. Duodecimo, pp. 264. Philadelphia: J. B. Lippincott Co. 1902. [$1.50.]

A book for the mother, with chapters on her care during pregnancy, parturition and the lying-in, and the care of the infant and young child. The author does not attempt to teach the mother too much, but gives only such advice as the intelligent mother will be able to profit by. M.J.F.

